# Impact of oral lipid and glucose tolerance tests on the postprandial concentrations of angiopoietin-like proteins (Angptl) 3 and 4

**DOI:** 10.1007/s00394-021-02748-0

**Published:** 2021-12-24

**Authors:** Andreas Schmid, Hannah Belikan, Alexandra Höpfinger, Andreas Schäffler, Thomas Karrasch

**Affiliations:** Department of Internal Medicine III, Giessen University Hospital, Klinikstraße 33, 35392 Giessen, Germany

**Keywords:** Angiopoietin-like protein 3, Angiopoietin-like protein 4, Oral lipid tolerance test, Oral glucose tolerance test, Adipocyte, Adipose tissue, Triglyceride

## Abstract

**Background:**

The postprandial regulation of angiopoietin-like proteins (Angptls) and their expression in adipocytes is poorly characterized.

**Objective:**

Circulating Angptl3 and 4 were analyzed in healthy individuals undergoing either an oral lipid tolerance test (OLTT; *n* = 98) or an oral glucose tolerance test (OGTT; *n* = 99). Venous blood was drawn after 0, 2, 4, and 6 h during OLTT and after 0, 1, and 2 h during OGTT. Anthropometric and laboratory parameters were assessed and concentrations of Angptls were quantified by enzyme-linked immunosorbent assay. Angptl gene expression in 3T3-L1 adipocytes and in murine adipose tissues and cellular fractions was analyzed by quantitative real-time PCR.

**Results:**

Angptl3 concentrations significantly decreased while Angptl4 levels continuously increased during OLTT. Both proteins remained unaffected during OGTT. Angptl3 and Angptl4 were expressed in murine subcutaneous and visceral AT with higher mRNA levels in mature adipocytes when compared to the stroma-vascular cell fraction. Both proteins were strongly induced during 3T3-L1 adipocyte differentiation and they were unresponsive to glucose in mature fat cells. Adipocyte Angptl3 (but not Angptl4) mRNA expression was inhibited by the polyunsaturated fatty acids arachidonic acid and docosahexaenoic acid, whereas nine types of dietary fatty acids remained without any effect.

**Conclusions:**

There is evidence of short-time regulation of Angptl3/4 levels upon metabolic stress. Angptl4 expression is high and Angptl3 expression is low in AT and restricted mainly to mature adipocytes without any differences concerning fat compartments. Whereas dietary fatty acids and glucose are without any effect, omega-3/-6-polyunsaturated fatty acids inhibited Anptl3 expression in adipocytes.

## Introduction

Besides its “classical” function in energy storage/release and as an isolating and structural tissue, investigations during the last three decades have led to an understanding of the adipose tissue as an active endocrine gland and an immunological organ substantially involved in inter-organ and inter-cellular communication [1–5]. These pleiotropic effects are mainly mediated by so-called adipokines, including a plethora of diverse adipocyte-secreted proteins and peptides exerting endocrine, paracrine, and autocrine effects [6–8].

The family of angiopoietin-like proteins (Angptls) is characterized by close structural relationship to the group of angiopoietins and comprises eight distinct polypeptides with differential tissue expression patterns [9]. In general, Angptls are secretory proteins sharing a common modular structure consisting of an N-terminal signal-peptide sequence, a coiled-coil domain, and a C-terminal fibrinogen-like domain, with the exception of Angptl8 [9, 10].

Angptl3 is mainly synthesized in liver [11, 12], whereas the expression sites of other Angptls are rather wide-spread, e. g. Angptl4 which also originates from adipose tissue [13–15]. Plasma Angptl3 is negatively regulated by the adipokine leptin and by insulin in mice [16]. It has an important role in the regulation of adipose tissue lipid metabolism and plasma free fatty acid levels (FFA), thereby acting as an hepatokine inducing lipolysis in adipocytes [17, 18]. Most importantly, Angptl3 represents a potent inhibitor of lipoprotein lipase (LPL), therefore being involved in the modulation of systemic triglycerides and lipoproteins [19, 20]. Of note, a cluster analysis of various adipokine serum levels identified Angptl3 as a factor related with parameters of obesity and inflammation [21]. Most recently, Angptl3 has been discussed as one of the most promising new cardiometabolic therapy targets [22].

Angptl4 was initially identified as a *fasting-induced adipose factor* (FIAF) and described as a target gene of *peroxisome-proliferator activated receptor gamma* (PPARγ) in adipose tissue [13, 23]. It is strongly induced during periods of caloric restriction and fasting in adipose tissue [24] and in the systemic circulation [25] as well as in skeletal muscle upon exercise [26]. Like Angptl3, it represents an inhibitor of LPL and is regarded as a crucial factor in systemic triglyceride and lipoprotein homeostasis [19]. While global Angptl4 deficiency results in increased visceral adipose tissue mass and inflammation in mice under high-fat diet (HFD) [27], adipose tissue specific Angptl4 depletion has beneficial effects on lipid metabolism, ectopic fat accumulation in liver and muscle and, glucose intolerance in HFD-fed animals [28]. Furthermore, on the level of central control of energy metabolism and nutritional behavior, Angptl4 is expressed in the hypothalamus under the control of appetite regulators and it mediates anorexigenic effects as an inhibitor of hypothalamic AMP-activated protein kinase (AMPK) [29].

The primary aim of the present study was to investigate the postprandial short-term dynamics of Angptl3 and Angptls4 during a standard oral glucose tolerance test (OGTT) vs. an oral lipid tolerance test (OLTT) in healthy individuals. Furthermore, murine cell lines and murine primary cells were used to investigate the impact of metabolic factors such as 11 fatty acid species and glucose on adipocyte Angptl3 and 4 mRNA expression and to identify potential molecular mediators standing behind the observed clinical observations.

## Material and methods

### Adipocyte cell culture and stimulation experiments

Murine 3T3-L1 pre-adipocytes [30] were cultured and differentiated into mature adipocytes as described previously [31]. Briefly, cells were cultured at 37 °C and 5% CO_2_ in Dulbecco’s Modified Eagle Medium (Biochrom AG, Berlin, Germany) supplemented with 10% newborn calf serum (Sigma-Aldrich, Deisenhofen, Germany) and 1% penicillin/streptomycin (Aidenbach, Germany) and were differentiated into adipocytes in DMEM/F12/glutamate medium (Lonza, Basel, Switzerland) supplemented with 20 µM 3-isobutyl-methyl-xanthine (Serva, Heidelberg, Germany), 1 µM corticosterone, 100 nM insulin, 200 µM ascorbate, 2 µg/mL transferrin, 5% fetal calf serum (FCS, Sigma-Aldrich, Deisenhofen, Germany), 1 µM biotin, 17 µM pantothenic acid, 1% penicillin/streptomycin (all from Sigma Aldrich, Deisenhofen Germany) and 300 µg/mL Pedersen-fetuin (MP Biomedicals, Illkirch, France) [32, 33]. A differentiation protocol reported in the literature [30, 34–37] was used with slight modifications and light-microscopy control of the cellular phenotype was done regularly. Lipid accumulation in adipocytes was additionally verified by Oil Red O staining of test samples. Mature adipocytes were incubated under serum-free conditions prior to stimulation experiments. FFA were purchased from Sigma-Aldrich (Deisenhofen, Germany) and dissolved in 10% BSA / EtOH in stock concentrations of 200 mM. Palmitic acid (PA; 100 µM), stearic acid (StA; 100 µM), margaric acid (MaA; 100 µM), myristic acid (MyA; 100 µM), lauric acid (LaA; 100 µM), oleic acid (OA; 10 µM), linoleic acid (LiA; 10 µM), palmitoleic acid (PoA; 10 µM), arachidonic acid (ArA; 10 µM), eicosapentaenoic acid (EPA; 10 µM), and docosahexaenoic acid (DHA; 10 µM) were applied in two separate overnight (18 h) stimulation experiments (*n* = 6 wells each). All the applied doses had been determined by previous experiments in adipocyte culture with respect to dose effects and cell viability [31, 38, 39]. Furthermore, LDH (lactate dehydrogenase) concentration was measured in the supernatants (Cytotoxicity Detection Kit, Roche, Mannheim, Germany) to exclude any unexpected cytotoxic effects.

### Isolation of mRNA and quantitative real-time PCR analysis of Angptl3 and 4 gene expression in murine adipose tissues and cells

Intra-abdominal and subcutaneous adipose tissue compartments were resected from wild-type C57BL/6 mice (bred under standard conditions and fed a normal chow) and sacrificed for tissue samples conformable to the German animal protection law (*§4 Abs. 3 Tierschutzgesetz*). A specific announcement was made at the local ethical committee (*Regierungspraesidium Giessen*: internal registration number: 544_M) that was approved subsequently. Small portions of fresh intra-abdominal and subcutaneous adipose tissue were digested with 0.225 U/mL of collagenase NB 6 (#17458, SERVA Electrophoresis; Heidelberg, Germany) and adipocytes were separated from stroma-vascular cells (SVC) via centrifugation (300 rcf, 10 min, 4 °C). Total mRNA was isolated from frozen murine total adipose tissues, from isolated mature adipocytes and SVC, and from cultured 3T3-L1 adipocytes as described previously [31]. Briefly, tissues were homogenized in TRIzol®-Reagent (Life Technologies GmbH, Darmstadt, Germany) in combination with gentleMACS dissociator and M-tubes (Miltenyi Biotec GmbH, Bergisch Gladbach, Germany) for dissociation and RNA was isolated applying RNeasy® Mini Kit (Qiagen, Hilden, Germany) including DNase digestion (RNase-Free DNase Set, Qiagen, Hilden, Germany).

For gene expression analysis, reverse transcription of RNA (QuantiTect Reverse Transcription Kit from Qiagen, Hilden, Germany) was performed to generate corresponding cDNA for real-time PCR (RT-PCR) (iTaq Universal SYBR Green Supermix, CFX Connect RT-PCR system; Bio-Rad, Munich, Germany). Expression levels of the target genes Angptl3 and 4 were normalized to the gene expression of glyceraldehyde-3-phosphate dehydrogenase (GAPDH) as a house-keeping gene applying the ΔΔC_T_ method. The primer-pairs used were:

Murine Angptl3: 5′-ACATGTGGCTGAGATTGCTGG-3′ / 5′-CCTTTGCTCTGTGATTCCATGTAG-3′

Murine Angptl4: 5′-ATGACTTCAGATGGAGGCTGG-3′ / 5′-AATTGGCTTCCTCGGTTCCC-3′.

Murine GAPDH: 5′-TGTCCGTCGTGGATCTGAC-3′ / 5′-AGGGAGATGCTCAGTGTTGG-3′

All oligonucleotides used were purchased from Metabion, Martinsried, Germany.

### Study cohorts

The study cohorts A (OLTT, oral lipid tolerance test) and B (OGTT, oral glucose tolerance test) were examined at the University Hospital of Regensburg, Germany, both including 100 healthy volunteers who gave their informed consent to the study approved by the local ethical committee (*Ethikkommission Universitätsklinikum Regensburg*, 11-101-0058 / 11-101-0068, date of approval: 2011-03-31). Both cohorts have been characterized in detail earlier by our group with respect to the regulation of the adipokine visfatin [40, 41] and baseline characteristics of both study populations have been published earlier [40, 41]. Briefly, study cohort A [40] consisted of 58 females and 42 males, 66 of them were normal weight and 34 overweight persons. Study cohort B [41] consisted of 64 females and 36 males, 51 of them were normal weight and 49 overweight individuals (all of them were insulin-sensitive with a normal HOMA index). Exclusion criteria were a positive history of any kind of illness, evidence of acute or chronic infection within 10 days prior to the OLTT or OGTT, age < 18 years or > 55 years, and any kind of medication except oral contraceptives. Pregnant and menstruating women were not admitted to the study. Anthropometric parameters such as age, BMI, hip circumference, waist circumference, waist/hip ratio, triceps skinfold thickness, and blood pressure were recorded. The patients’ history concerning type 2 diabetes and cardiovascular diseases as well as habits such as smoking and hormonal contraception were documented.

### Study cohort A: oral lipid tolerance test (OLTT)

As reported earlier [41], a preparation was used that was completely free of proteins and carbohydrates but contained all classes of fatty acid species (saturated, monounsaturated and polyunsaturated fatty acids). Briefly, the OLTT solution (160 mL; 758.1 kcal; 75 g vegetable fat as triglycerides, 9.2 g fatty acids as pure vegetable oils) comprised the following three components: *Component 1:* 150 mL water/fat solution (Calogen^R^ NUTRICIA-Neutral, Pfrimmer Nutricia, Erlangen, Germany): 75 g vegetable fat as pure triglycerides containing 7.95 g saturated fatty acids, 45.6 g monounsaturated fatty acids, 21.45 g polyunsaturated fatty acids, 69 g water, 0.15 g carbohydrates (insignificant trace amount), 675 kcal. *Component 2:* 5 mL (4.6 g; 41.5 kcal) sun flower oil containing 10.2% saturated fatty acids, 26.3% monounsaturated fatty acids, and 63.5% polyunsaturated fatty acids. *Component 3:* 5 mL (4.6 g; 41.6 kcal) olive oil containing 14% saturated fatty acids, 78% monounsaturated fatty acids, and 8% polyunsaturated fatty acids. The study procedure started after an overnight fast of at least 12 h. During the observation time, the volunteers had to rest and not to eat or smoke. The ingestion of 500 mL of pure water was allowed over the 6 h. Venous blood samples were drawn immediately following the lipid ingestion and after 2, 4, and 6 h. Blood serum was prepared from the samples by 10 min of centrifugation at 4000 rotations per min (rpm) and 4 °C.

### Study cohort B: oral glucose tolerance test (OGTT)

As reported earlier [41], all volunteers underwent a standard 75 g, 2 h OGTT between 8:00 am and 10:00 am after an overnight fast of 12 h. Venous blood samples were drawn at 0, 60 and 120 min and serum was prepared as described above (10 min, 4000 rpm, 4 °C).

### Quantification of serum Angptl concentrations

Concentrations of serum Angptl3 and Angptl4 were measured in duplicates by ELISA techniques (DuoSet ELISA development systems, R&D Systems, Wiesbaden, Germany) and are expressed as means ± standard deviation. Measurement of plasma glucose, plasma insulin, C-reactive protein (CRP), total cholesterol, triglycerides, high-density lipoprotein cholesterol (HDL), and low-density lipoprotein cholesterol (LDL) was performed by standard techniques at the Institute of Clinical Chemistry and Laboratory Medicine, University of Regensburg, Germany, as described earlier [41].

### Statistical analysis

For explorative data analysis, a statistical software package (*SPSS 27.0*) was used. Angptl serum concentrations and gene expression levels did not follow a *Gaussian* distribution. Non-parametric numerical parameters were analyzed by the *Mann–Whitney U*-test (for 2 unrelated samples), the *Kruskal–Wallis* test (> 2 unrelated samples), the *Wilcoxon* test (for 2 related samples) or the *Friedman* test (> 2 related samples). Correlation analysis was performed using the *Pearson* test (parametric parameters) or the *Spearman* test (non-parametric parameters). Partial correlation analysis was applied to control for possible covariates. A p-value below 0.05 (two tailed) was considered as statistically significant. In the figures, the bars are showing the mean values and the whiskers are giving the SEM (standard error of the mean).

## Results

### Sexual dimorphism and correlations of basal Angptl3 and Angptl4 serum concentrations

The characteristics of the two study populations are shown in Table 1. Angplt3 and 4 could be measured successfully in duplicate in 98 participants of the OLTT study group (57 females, 41 males; mean age: 28.3 ± 7.7 years; mean BMI: 24.16 ± 5.06 kg/m^2^). Overall mean Angptl4 serum levels were calculated as 252 ± 159 ng/mL. Females had significantly higher concentrations when compared to male subjects (286 ± 185 ng/mL versus 206 ± 98 ng/mL; *p* = 0.014). In contrast, there was no significant gender-specific difference in Angptl3 concentrations (mean: 111 ± 62 ng/mL).

**Table 1:** Characteristics of the study populations (**A**) and correlation analysis of baseline Angptl3 (**B**) and Angptl4 (**C**) serum concentrations with anthropometric and biochemical parameters

	OLTT (* n* = 98)		OGTT (* n* = 99)	
A. Characteristics of the study populations				
Males (n)	41		35	
Females (n)	57		64	
Age (years)	28.3 ± 7.7		26.7 ± 6.2	
BMI (kg/m^2^)	24.16 ± 5.06		24.91 ± 5.02	
Angptl3 (ng/ml)	111 ± 62		111 ± 52	
Angptl4 (ng/ml)	252 ± 159		266 ± 136	
B. Correlation of Angptl3 with anthropometric and biochemical parameters				
Age	rho = + 0.108	*p* = 0.289	rho = + 0.214	*p* = 0.034
WHR	rho = + 0.040	*p* = 0.693	rho = + 0.079	*p* = 0.437
Skinfold thickness	rho = − 0.107	*p* = 0.295	rho = − 0.078	*p* = 0.441
Insulin	rho = − 0.191	*p* = 0.060	rho = − 0.118	*p* = 0.246
CRP	rho = − 0.005	*p* = 0.960	rho = − 0.165	*p* = 0.102
Triglycerides	rho = − 0.093	*p* = 0.363	rho = − 0.196	*p* = 0.051
HDL	rho = − 0.151	*p* = 0.138	rho = − 0.108	*p* = 0.285
Angptl4	rho = − 0.088	*p* = 0.391	**rho = **− **0.371**	*p*** < 0.001**
RBP4	rho = + 0.151	*p* = 0.138	**rho = **− **0.415**	*p*** < 0.001**
FABP4	rho = − 0.049	*p* = 0.633	rho = − 0.140	*p* = 0.187
C. Correlation of Angptl4 with anthropometric and biochemical parameters				
Age	rho = + 0.008	*p* = 0.937	**rho = **− **0.222**	*p*** = 0.027**
WHR	rho = − 0.149	*p* = 0.144	**rho = **− **0.269**	*p*** = 0.007**
Skinfold thickness	**rho = + 0.236**	*p*** = 0.019**	**rho = + 0.293**	*p*** = 0.003**
Insulin	rho = + 0.054	*p* = 0.598	rho = + 0.085	*p* = 0.403
CRP	**rho = + 0.325**	*p*** = 0.001**	**rho = + 0.251**	*p*** = 0.012**
Triglycerides	rho = + 0.004	*p* = 0.972	rho = + 0.052	*p* = 0.608
HDL	**rho = + 0.220**	*p*** = 0.030**	**rho = 0.383**	*p*** < 0.001**
Angptl3	rho = − 0.088	*p* = 0.391	**rho = **− **0.371**	*p*** < 0.001**
RBP4	rho = + 0.044	*p* = 0.667	**rho = + 0.297**	*p*** = 0.003**
FABP4	**rho = + 0.234**	*p*** = 0.022**	**rho = + 0.254**	*p*** = 0.015**

*Angptl3* angiopoietin-like protein 3, *Angptl4* angiopoietin-like protein 4, *CRP* C-reactive protein, *FABP4* fatty acid binding protein 4, *HDL* high-density lipoprotein, *OGTT* oral glucose tolerance test, *OLTT* oral lipid tolerance test, *RBP4* retinol binding protein 4, *WHR* waist-hip ratio

Within the OGTT study group, Angpt3 and 4 could successfully be measured in duplicate in 99 individuals (64 females, 35 males) with a mean age of 26.7 ± 6.2 and mean BMI of 24.97 ± 5.02 kg/m^2^. Mean baseline Angptl4 concentrations were 266 ± 136 ng/mL. Of note, a highly significant sexual dimorphism with elevated Angptl4 concentrations in females was also found within this study cohort (304 ± 150 ng/mL versus 198 ± 67 ng/mL; *p* < 0.001). Mean Angptl3 concentrations were 111 ± 52 ng/mL. Slightly elevated levels of Angptl3 were found in males (127 ± 53 ng/mL versus 101 ± 49 ng/mL in females). However, this difference is marginal and of questionable significance (*p* = 0.028). Spearman-rho test was applied to identify multiple correlations of baseline Angptl3 and 4 levels with anthropometric and biochemical parameters. The results are summarized in Table 1. In the OGTT cohort, retinol binding protein (RBP) 4 was negatively correlated with Angptl3 (rho = − 0.415, *p* < 0.001) and was positively correlated with Angptl4 (rho =  + 0.297, *p* = 0.003). Of note, significant positive correlations of Angptl4 with fatty acid binding protein (FABP) 4 were observed in both study cohorts. Despite some further interesting but solely correlative associations, the most robust and significant parameter in both cohorts is the above mentioned sexual dimorphism of Angptl4 levels. Of note, Angptl4 levels were found to be positively correlated with skinfold thickness (rho =  + 0.288, *p* < 0.001) and negatively correlated with waist-hip ratio (WHR) (rho = − 0.196, *p* = 0.006) in the overall study cohort (OGTT + OLTT cohort, *n* = 197). Since skinfold thickness was higher (17.2 ± 10.1 mm versus 8.3 ± 7.3 mm; *p* < 0.001) and WHR was lower in women when compared to men (0.82 ± 0.08 versus 0.90 ± 0.08; *p* < 0.001), both parameters might contribute to the observed sex-dependent differences in circulating Angptl4 concentrations.

### Circulating Angptl3 and Angptl4 levels are differentially regulated by oral lipid ingestion but not by oral glucose uptake

**Fig. 1:**
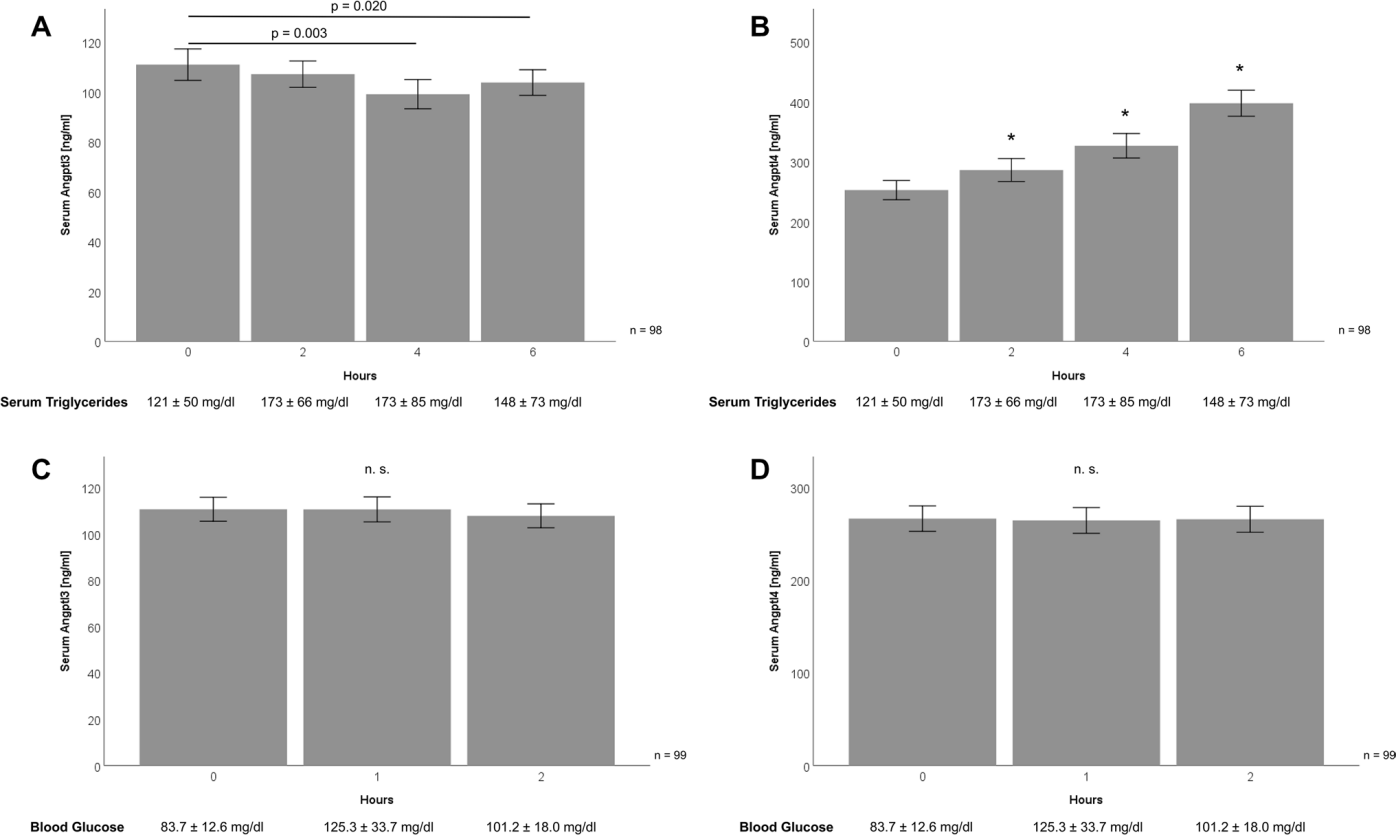
Circulating levels of Angptl3 and Angptl4 are differentially regulated upon lipid ingestion (OLTT) but not by glucose uptake (OGTT). OLTT participants orally received a lipid preparation (160 mL; 758.1 kcal; 75 g vegetable fat as triglycerides, 9.2 g fatty acids as pure vegetable oils) and venous blood was drawn at baseline (0 h) and after 2, 4, and 6 h (**A**, **B**). During OGTT, blood was sampled 0, 1, and 2 h after oral uptake of 75 g glucose (**C**, **D**). Raise of serum triglycerides during OLTT (**A**, **B**) and of blood glucose levels during OGTT (**C**, **D**) is illustrated by means ± standard deviation for the examined time-points. Angptl3 and Angptl4 concentrations in blood serum were measured by ELISA. *Angptl3* angiopoietin-like protein3, *Angptl4* angiopoietin-like protein4, *ELISA* enzyme-linked immunosorbent assay, *OGTT* oral glucose tolerance test, *OLTT* oral lipid tolerance test

Baseline serum concentrations of Angptl3 were 111.1 ± 62.1 ng/mL and there was a slight but significant decrease upon lipid ingestion after 4 h (*p* = 0.003) and 6 h (*p* = 0.02) (Fig. 1A). On the contrary, Angptl4 levels continuously increased in a stepwise manner upon lipid uptake from basal 252.5 ± 158.8 ng/mL up to 397.7 ± 215.8 ng/mL after 6 h (*p* < 0.001) (Fig. 1B). This increase during OLTT was observed for male (*n* = 41, *p* < 0.001) as well as for female subjects (*n* = 57, *p* < 0.001). Oral glucose uptake did not affect Angptl3 and Angptl4 concentrations in the whole study cohort (Fig. 1C, D) as well as in the subgroups of male and female subjects.

In both study cohorts, baseline Angptl3 and Angptl4 serum levels were not correlated with BMI and did not differ in normal weight (NW; BMI < 25 kg/m^2^) and overweight (OW; BMI >  = 25 kg/m^2^) study participants (data not shown). Oral glucose ingestion did not result in significant changes of Angptl levels in NW and OW subjects whereas Angptl4 concentrations considerably increased during OLTT in both subgroups (*p* < 0.001). While oral lipids induced a modest yet significant decline of Angptl3 in NW individuals (*n* = 65; *p* < 0.001), this effect was absent in OW subjects (*n* = 33; *p* = 0.159).

### Angptl3 and 4 gene expression is strongly induced during adipocyte differentiation

**Fig. 2:**
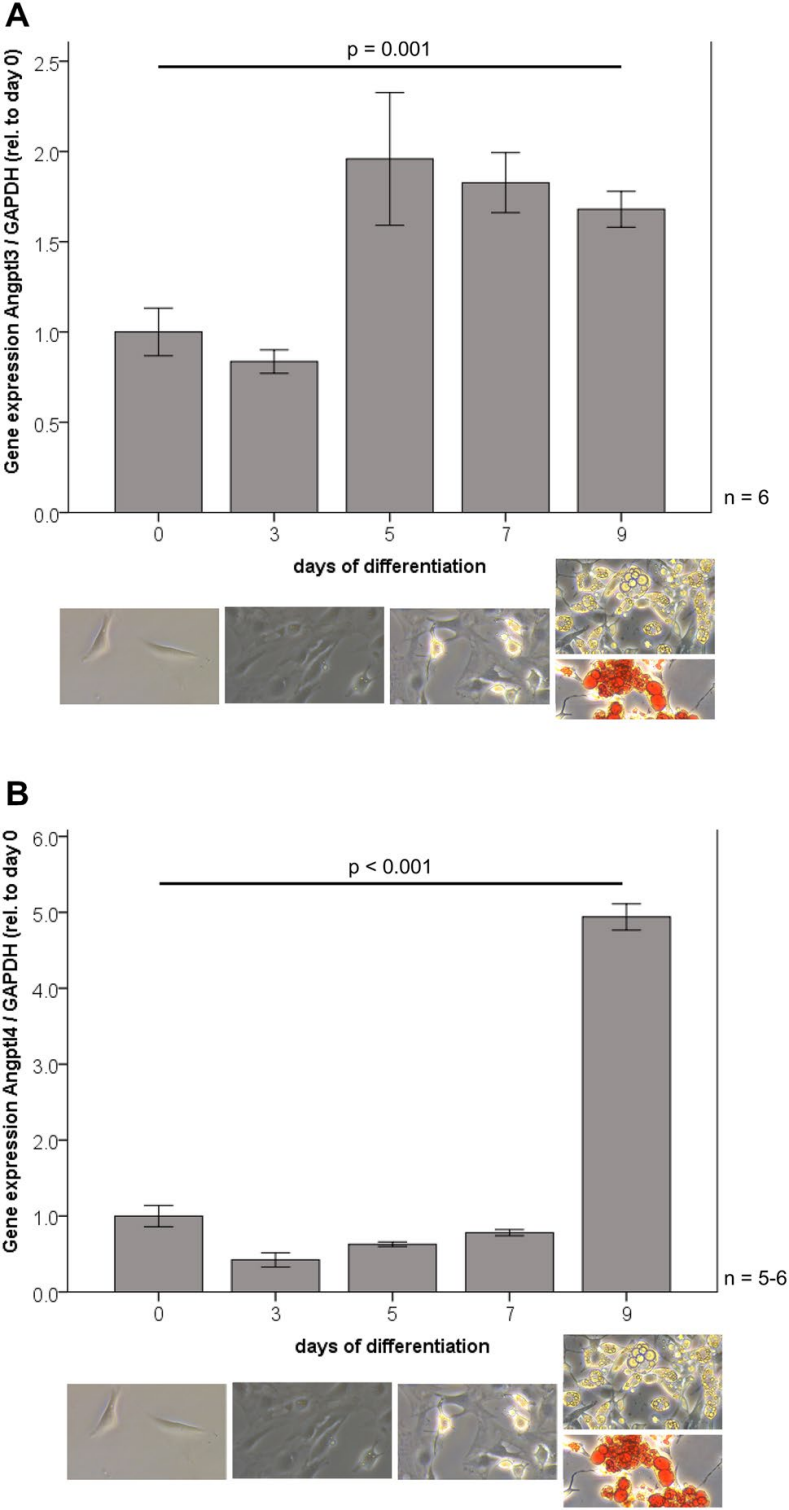
Angptl3 (**A**) and Angptl4 (**B**) gene expression is induced during adipocyte differentiation. During hormonally induced differentiation of murine 3T3-L1 fibroblast-like pre-adipocytes into mature adipocytes, mRNA levels of Angptl3 and Angptl4 were significantly upregulated during late stages of the differentiation process (day 0–3: undifferentiated, fibroblast-like pre-adipocytes; day 3–5: ongoing differentiation and lipid accumulation; day 7–9: late differentiation with fully differentiated and mature adipocytes showing a round shape and intensive lipid accumulation a day 7). Cellular phenotypes during differentiation were illustrated by representative light-microscopy images (magnification: × 200) and lipid accumulation in mature adipocytes was visualized by Oil Red O staining. Angptl3 mRNA expression was quantified by real-time PCR and expression levels were related to GAPDH expression (house-keeping gene). *Angptl3* angiopoietin-like protein 3, *Angptl4* angiopoietin-like protein 4, *GAPDH* glyceraldehyde-3-phosphate dehydrogenase

Angptl3 and Angptl4 mRNA levels were investigated during hormonally induced 3T3-L1 adipocyte differentiation. As shown in Fig. 2A, Angptl3 gene expression was significantly induced at day 5 of adipocyte differentiation and remained elevated during late adipocyte differentiation and in mature adipocytes at day 9 (*p* = 0.001). Angptl4 mRNA expression (Fig. 2B) was low in pre-adipocytes and remained on low levels until the final stage of adipocyte differentiation (day 7–9). In mature and fully differentiated adipocytes (day 9), Angptl4 was strongly increased ~ fivefold (*p* < 0.001).

### Angptl3 and 4 gene expression is not different between intra-abdominal and subcutaneous compartments of total adipose tissue in mice

**Fig. 3:**
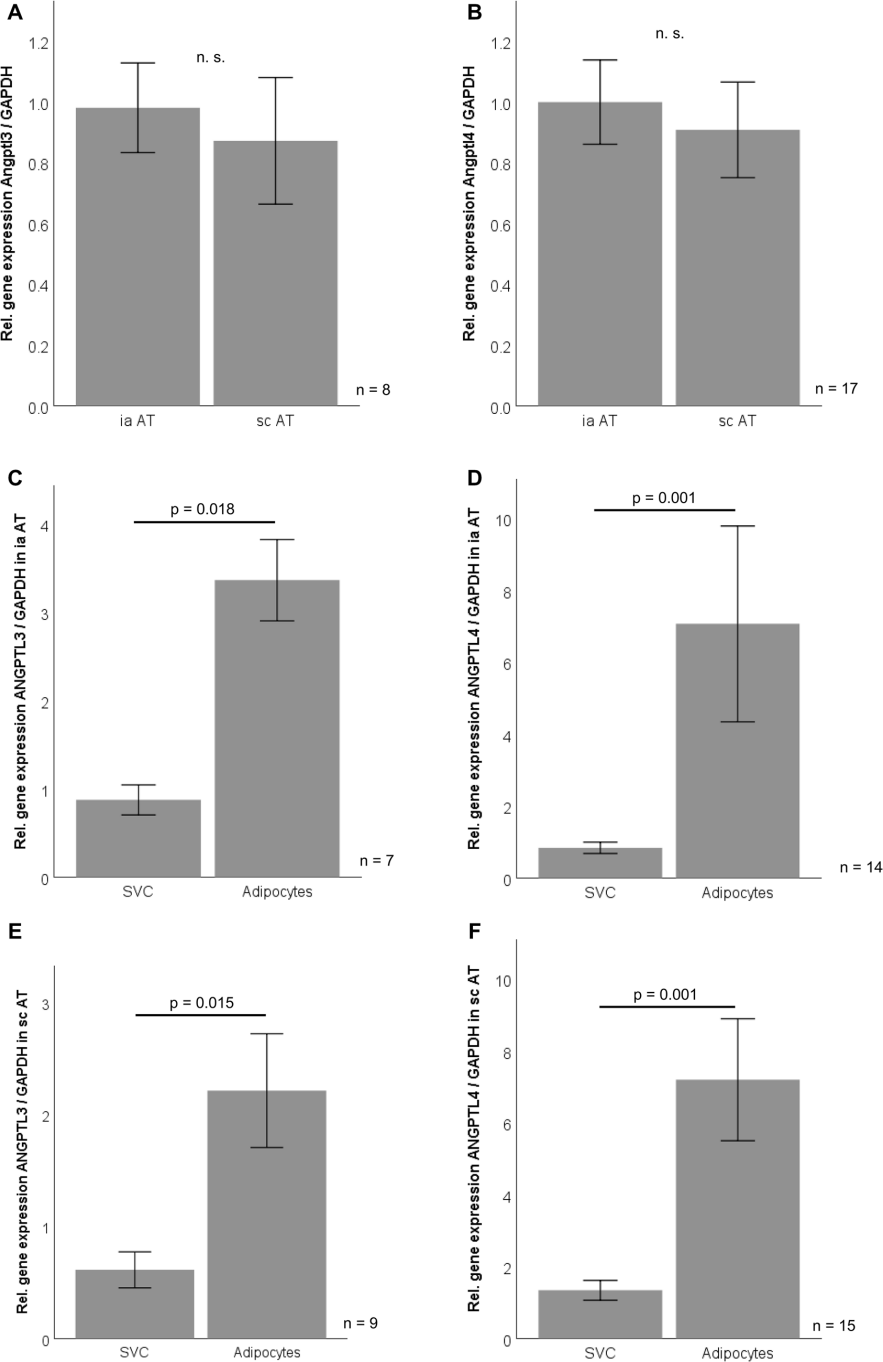
Quantification of Angptl3 and Angptl4 mRNA expression in different adipose tissue compartments and cellular fractions. Gene expression of Angptl3 and Angptl4 is not different between total intra-abdominal (ia) and total subcutaneous (sc) adipose tissue (AT) resected from C57BL/6 wild-type mice (**A**, **B**). Angptl3 and Angptl4 expression is abundant in mature adipocytes but not in the stroma-vascular cell (SVC) fraction isolated from intra-abdominal (**C**, **D**) and subcutaneous (**E**, **F**) adipose tissue. Angptl3, angiopoietin-like protein 3; Angptl4, angiopoietin-like protein 4

Gene expression analysis was performed in intra-abdominal and subcutaneous adipose tissue specimens (total adipose) obtained from C57BL/6 wild-type mice. As summarized in Fig. 3A, B, there were no significant differences in Angptl3 and Angptl4 mRNA expression between both fat compartments. In general, expression levels of Angptl3 in adipose tissue were lower (mean *C*_T_ = 27.5 ± 1.1 cycles) than Angptl4 levels (mean *C*_T_ = 23.3 ± 0.9 cycles).

### Angptl3 and 4 gene expression is abundant in mature adipocytes but very low in the stroma-vascular cell fraction

Subsets of adipose-resident cell-types (mature adipocytes vs. cells of the stroma-vascular fraction (SVC)) were isolated from intra-abdominal and subcutaneous adipose tissue and analyzed for Angptl3/4 expression (Fig. 3C–F). Most interestingly, the expression of Angptl3 and Angptl4 was significantly higher in mature adipocytes when compared to cells of the SVC fraction in both compartments of adipose tissue (intra-abdominal and subcutaneous). There were no significant differences between intra-abdominal and subcutaneous compartments concerning Angptl3 gene expression in adipocytes and SVC. Of note, subcutaneous adipocytes exhibited higher Angptl4 expression (*p* = 0.023) than intra-abdominal adipocytes, whereas no differences were detected for SVC.

### Effects of fatty acids and glucose on adipocyte Angptl3 and Angptl4 mRNA expression in vitro

**Fig. 4:**
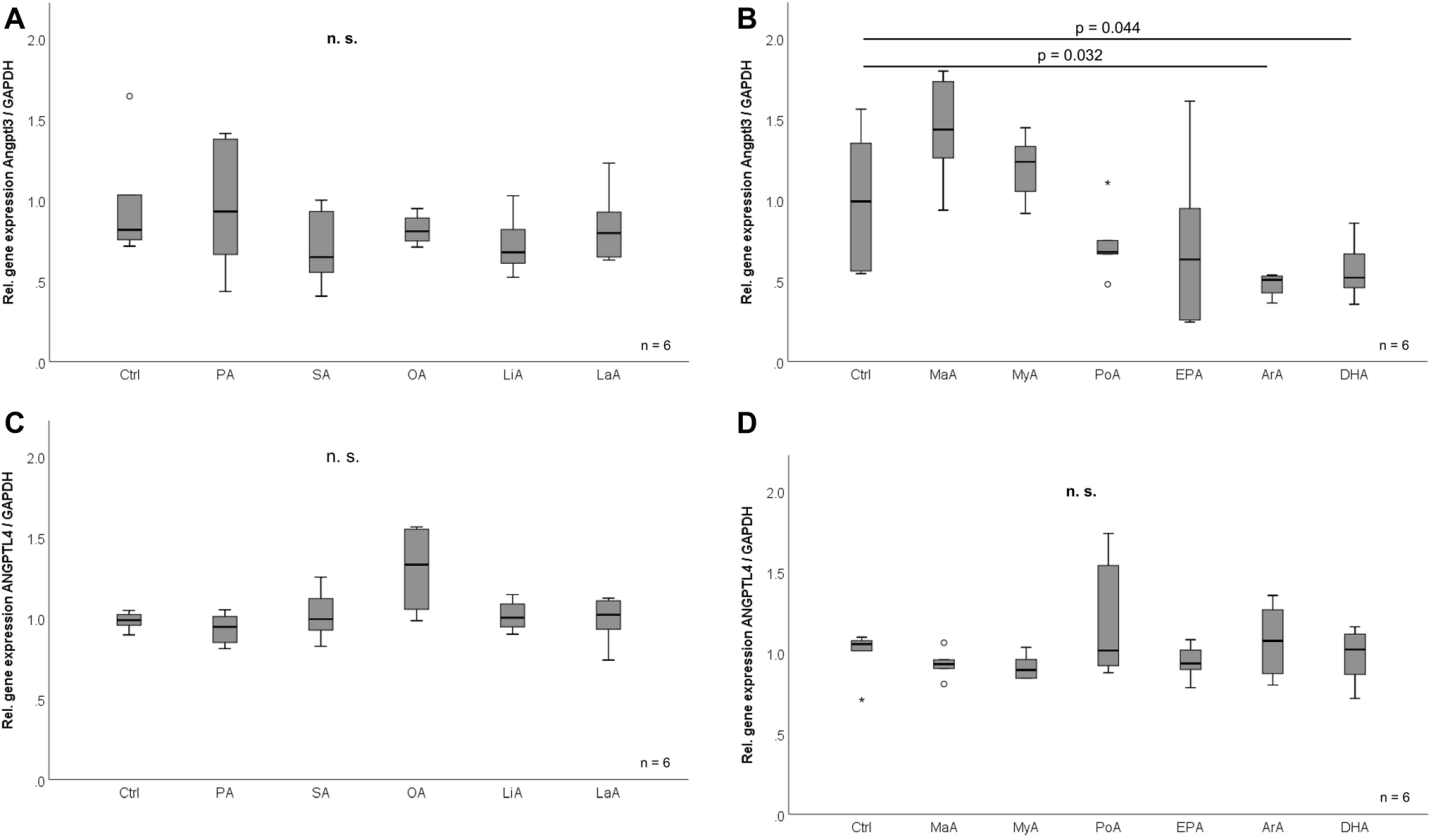
Effects of fatty acids on adipocytic Angptl3 and Angptl4 gene expression. 3T3-L1 adipocytes were treated with a broad panel of 11 *saturated* (lauric acid (LaA), 100 µM); margaric acid (MaA), 100 µM; myristic acid (MyA), 100 µM; palmitic acid (PA), 100 µM; stearic acid (SA), 100 µM) as well as *monounsaturated* (oleic acid (OA), 10 µM) and *polyunsaturated* fatty acids (arachidonic acid (ArA), 10 µM; docosahexaenoic acid (DHA), 10 µM; eicosapentaenoic acid (EPA), 10 µM; linoleic acid (LiA), 10 µM; palmitoleic acid (PoA), 10 µM) (**A**–**D**). Cells were incubated with free fatty acids for 18 h prior to RNA isolation and RT-PCR analysis. *Angptl3* angiopoietin-like protein 3, *Angptl4* angiopoietin-like protein 4, *Ctrl* control, *n.s.* not significant

A broad panel of 11 fatty acids was tested for their potential impact on Angptl3/4 expression in 3T3-L1 adipocytes (Fig. 4A–D). Most of the dietary fatty acid species—saturated as well as unsaturated fatty acids—did not affect Angptl3/4 expression (Fig. 4A, B). Of note, the polyunsaturated, membrane-derived, pro-inflammatory arachidonic acid (ArA) and the metabolically protective docosahexaenoic acid (DHA) significantly reduced Angptl3 mRNA expression (Fig. 4B). No significant effects of fatty acids were observed regarding Angptl4 mRNA levels (Fig. 4C, D). In the supernatants of mature 3T3-L1 adipocytes, high (25 mM) vs. low-normal (5.6 mM) glucose concentrations were applied to investigate effects on Angptl expression. The basal expression levels of Angptl3 and Angptl4 were not affected by high glucose concentrations (data not shown).

## Discussion

In both clinical study cohorts investigated, females had significantly higher Angptl4 serum concentrations when compared to males. Interestingly, our data suggest that this observation might—at least in part—be due to a higher proportion of subcutaneous adipose tissue (indicated by skinfold thickness) and lower proportion of visceral adipose tissue (lower waist-hip ratio) in females in the present study. The significant correlations of skinfold thickness and WHR with circulating Angptl4 levels as detected in this study cohort are novel and should further be evaluated.

To the best of our knowledge, this is the first study showing significantly higher Angptl4 serum concentrations in fasting and healthy females [42]. Higher Angptl4 concentrations have been reported in patients on hemodialysis [43] and in patients suffering from rheumatoid arthritis [44]. Interestingly, a clinical trial investigating the effect of ethinyl estradiol-cyproterone acetate in adolescent girls with androgen excess reported that the expression of Angptl4 in subcutaneous adipose tissue significantly increased upon hormonal treatment [45]. Thus, future in vitro studies are necessary to investigate a putative and local effect of estradiol, progesterone, and also testosterone on Angptl4 gene expression in subcutaneous adipose tissue.

Our findings from two clinical study cohorts of relatively young and healthy volunteers illustrate the response of circulating Angptl3 and Angptl4 concentrations to metabolic stress. During an oral lipid tolerance test over 6 h applying a carbohydrate- and protein-free, vegetable-derived triglyceride solution containing high proportions of mono- and polyunsaturated fatty acids from plant oils, we observed a significant decline of serum Angptl3 concentrations. Even more impressing, we could demonstrate in parallel a stepwise and highly significant upregulation of Angptl4 concentrations. To the best of our knowledge, this is the first study in a large cohort of healthy individuals reporting this kind of postprandial short-time regulation in vivo. Since Angptl4 has been described as a fasting-induced adipokine and hepatokine inhibiting triglyceride uptake and storage in adipocytes [19], this observed upregulation upon oral triglyceride ingestion might be unexpected. Increased Angptl4 serum levels have been associated with impaired clearance of circulating triglycerides due to inhibition of lipoprotein lipase (LPL) [46]. On the other hand, Angptl4 depletion causes elevated LPL activity and low circulating triglyceride levels [47]. Moreover, Angptl4 expression in adipose tissue is decreased in the postprandial compared to fasting state in obese individuals [48].

Taken together, oral lipid tolerance tests should be investigated in obese and type 2 diabetic patients to compare the dynamics of Angptl3 and Angptl4 after lipid uptake. Elaborating on the present findings for metabolically healthy, normal weight and overweight patients, these future study designs will allow to reveal potential correlations of BMI and/or insulin resistance with regulatory effects of nutritional lipids. The involvement of Angiopoietin-like proteins in the regulation of lipid metabolism is rather complex, since Angptl8 activates Angptl3 and decreases Angptl4-mediated inhibition of LPL [19, 49]. Thus, the impact of Angptl4 on postprandial lipid partitioning does not exclusively depend on the quantity of circulating concentrations. Furthermore, a lipid-induced increase of Angptl4 levels can be considered as part of a potential regulatory/compensatory mechanism due to elevated postprandial Angptl8 concentrations [49]. Interestingly, a recent study reported a postprandial decline of systemic Angptl3 and Angptl8 levels in females after a 7-day diet rich in polyunsaturated fatty acids [50].

Furthermore, the molecular composition of the triglyceride solution ingested (in our study > 85% mono- and polyunsaturated fatty acids) plays an important role. Most studies use dietary protocols or mixed meal tests containing relatively undefined compositions of fatty acids and containing also proteins and carbohydrates. The present study was designed to dissect carbohydrate-induced from lipid-induced effect and to characterize to potential role of 11 fatty acids on adipocyte Angptl3/4 expression. Whereas glucose had no effects in vivo and in vitro, the effects of lipid uptake in vivo and of fatty acids in vitro were of differential nature. Surprisingly, none of the classical 9 dietary saturated and unsaturated fatty acids affected Angptl3/4 gene expression in adipocytes. Of course, it cannot be excluded that hepatic expression of these Angptls [51] might occur in reaction to these fatty acids. Thus, future studies should investigate effects of FFA and/or triglycerides on hepatocytes as well.

Of note, the ω-6-polyunsaturated, membrane-derived, pro-inflammatory arachidonic acid and the ω-3-polyunsaturated, membrane- and nutritional-derived, cardio-metabolically protective docosahexaenoic acid (DHA) significantly downregulated Angptl3 mRNA expression. It seems fascinating to speculate that the metabolically protective effects of DHA might be mediated by inhibition of adipocyte-derived Angpt3 expression. The molecular consequence would comprise a disinhibition of LPL-mediated lipolysis and a consecutive reduction of triglycerides. We could demonstrate that long-chain polyunsaturated fatty acids such as DHA (omega-3) and arachidonic acid (omega-6) are able to decrease Angptl3 expression. This seems surprising from a first point of view, since arachidonic acid is commonly discussed as a more pro-inflammatory fatty acid whereas DHA is commonly interpreted to act as a more anti-inflammatory factor. Angptl3 is commonly related to obesity, lipolysis (it acts as an inducer of lipolysis) and inflammation. Insulin inhibits lipolysis and Angptl3 expression as well. The disinhibition of lipolysis commonly seen in obesity-related insulin resistance (a condition characterized by high insulin levels that fail to exert their action due to receptor resistance) could be mediated - at least in part—by reduced Angptl3 concentrations. Up to now, it remains an unsolved question why different PUFAs exert differential effects on the regulation of Angptl3 expression. Future molecular studies have to address this question in adipocytes. However, a differential involvement and cross-talk of Angptl3 with fatty acids in the context of lipolysis, lipoprotein lipase pathway, insulin resistance, and inflammation is highly probable.

Interestingly, the present study identified significant positive correlations of Angptl4 with FABP4 and RBP4, whereas Angptl3 was negatively correlated with RBP4 in the OGTT study cohort. Since both FABP4 and RBP4 represent lipocalins with functions in lipid transport and are associated with glucose metabolism and insulin sensitivity/resistance [52], these findings might indicate a mechanism especially of triglyceride-inducible systemic Angptl4 linking lipid with carbohydrate metabolism. This issue deserves further attention and should also be addressed by future studies involving OLTT in obese and diabetic individuals as suggested above.

Gene expression analysis performed in murine adipose tissues revealed comparable Angptl3 and Angptl4 mRNA levels in subcutaneous and intra-abdominal compartments. Importantly, we found a predominant expression in mature adipocytes when compared to stroma-vascular cells. Furthermore, a strong induction of Angptl3 and 4 gene expression levels during differentiation of murine 3T3-L1 fibroblasts into mature adipocytes was observed. Our data on the expression of Angptl4 are in accordance with findings reported by earlier studies [53, 54] but give a more differentiated and detailed expression profile. In contrast, the expression of Angptl3 in adipocytes is detectable but might play a minor role when compared to the liver as the predominant source [51]. Thus, some Angptl3-induced effects on adipocyte lipid metabolism [17] might—at least in part—be mediated in an auto- or paracrine rather than an endocrine manner.

It is a limitation of the present study that no protein data are available (e. g. Western blot analysis or ELISA measurements in cell culture supernatants). Future work has to be done in order to verify the observed effects on gene expression on the level of the respective protein expression.

## Conclusions

The study proves the existence of short-time regulation and short-time dynamics of systemic Angptl3/4 levels upon metabolic stress in vivo. Angptl4 expression is high and Angptl3 expression is low in adipose tissue and restricted mainly to mature adipocytes without any differences concerning fat compartments. Whereas dietary (nutritional) fatty acids and glucose did not regulate Angptl3/4 expression in adipocytes, omega-3 and omega-6-polyunsaturated fatty acids inhibited Angptl3 gene expression by yet unknown mechanisms. Future studies are necessary to dissect hepatic from adipocyte sources of Angptl3/4 concerning their secretion upon oral lipid uptake. Furthermore, the physiological significance and the mechanism of arachidonic- and DHA-induced effects on Angptl3 expression in adipocytes have to be investigated.
